# Determination of MIC Quality Control Ranges for Ceftibuten-Avibactam (Fixed 4 μg/mL), a Novel β-Lactam/β-Lactamase Inhibitor Combination

**DOI:** 10.1128/jcm.01657-22

**Published:** 2023-07-03

**Authors:** Michael D. Huband, Kelley A. Fedler, Helio S. Sader, Gregory G. Stone, Mariana Castanheira

**Affiliations:** a JMI Laboratories, North Liberty, Iowa, USA; b Pfizer Inc., New York, New York, USA; Pattern Bioscience

**Keywords:** ceftibuten, avibactam, M23, quality control

## Abstract

Ceftibuten/ARX-1796 (avibactam prodrug) is a novel oral antibacterial combination in early clinical development for the treatment of complicated urinary tract infections (cUTI) including pyelonephritis. ARX-1796 is the novel avibactam prodrug being combined with ceftibuten for oral dosing that is converted to active avibactam *in vivo*. A Clinical and Laboratory Standards Institute (CLSI) M23 (2018) tier 2 broth microdilution quality control (QC) study was conducted with ceftibuten-avibactam to establish MIC QC ranges. Ceftibuten-avibactam broth microdilution QC ranges were approved for Escherichia coli ATCC 25922 (0.016/4 to 0.12/4 μg/mL), E. coli NCTC 13353 (0.03/4 to 0.12/4 μg/mL), Klebsiella pneumoniae ATCC 700603 (0.06/4 to 0.25/4 μg/mL), K. pneumoniae ATCC BAA-1705 (0.03/4 to 0.25/4 μg/mL), and K. pneumoniae ATCC BAA-2814 (0.12/4 to 0.5/4 μg/mL) by the CLSI Subcommittee on Antimicrobial Susceptibility Testing in January 2022. Approved ceftibuten-avibactam QC ranges will support future clinical development, device manufacturers, and routine patient care.

## INTRODUCTION

The Centers for Disease Control and Prevention (CDC) describes carbapenem-resistant *Enterobacterales* (CRE) as urgent threats and extended-spectrum β-lactamase (ESBL)-producing *Enterobacterales* as serious threats, according to the 2019 CDC Antibiotic Resistance Threats in the United States report (1). Similarly, the 2017 World Health Organization global priority list of antibiotic-resistant bacteria describes CRE and ESBL-producing *Enterobacterales* as priority 1 critical threats (2). The β-lactamases produced by these drug-resistant *Enterobacterales* isolates often reside on mobile elements that are easily transmitted between species. The emergence and spread of CTX-M-type (ESBL) β-lactamases and KPC carbapenemases have created the need for new and more effective antibacterial agents, including new β-lactam/β-lactamase inhibitor combinations (3, 4). Gram-negative bacterial resistance has also increased in urinary tract infections, conferring resistance to conventional oral antibacterials, including ciprofloxacin, nitrofurantoin, and trimethoprim-sulfamethoxazole (5).

Ceftibuten is a semisynthetic third-generation oral cephalosporin approved by the United States Food and Drug Administration in 1995 for the treatment of acute bacterial exacerbations of chronic bronchitis, acute bacterial otitis media, and pharyngitis and tonsillitis caused by indicated organisms (6). Ceftibuten has also been used clinically in the treatment of complicated and uncomplicated urinary tract infections, although these are not approved indications (7, 8). The effectiveness of cephalosporins, including ceftibuten, against *Enterobacterales* isolates has diminished due to the emergence and spread of ESBL- and carbapenemase (KPC and OXA-48)-producing isolates. Ceftibuten/ARX-1796 (avibactam prodrug) is a new orally active antibacterial combination in early clinical development possessing potent *in vitro* activity against ESBL-, KPC-, OXA-48-, and AmpC-producing *Enterobacterales* isolates, including those causing urinary tract infections (9–11; H. S. Sader, C. G. Carvalhaes, M. D. Huband, R. E. Mendes, and M. Castanheira, 12). ARX-1796 is the novel prodrug of avibactam being combined with ceftibuten for oral dosing that is converted to active avibactam *in vivo*.

In this study, we performed CLSI M23 (13) tier 2 multilaboratory quality control (QC) testing to establish broth microdilution QC ranges for ceftibuten-avibactam and add additional QC ranges for ceftibuten and ceftazidime-avibactam (fixed 4 μg/mL).

## MATERIALS AND METHODS

### Broth microdilution.

This broth microdilution quality control study for ceftibuten-avibactam followed CLSI M23 (13) tier 2 guidelines for studies requiring a multilaboratory investigation. Concurrent testing of ceftibuten and ceftazidime-avibactam was included as an experimental control. Eight qualified laboratories contributed MIC results for this study, each representing a separate and distinct institution (Table 1). Each participating laboratory utilized 3 lots of cation-adjusted Mueller-Hinton broth (CAMHB) meeting ISO/TS 16782:2016 criteria (14) obtained from 3 different commercial manufacturers (13). Commercial medium manufacturers consisted of Difco (Detroit, MI, USA; lot 9156821, medium A), BD (BBL; Sparks, MD, USA; lot 0252344, medium B), and Oxoid (Hampshire, UK; lot 3163254, medium C). Frozen-form MIC panels (lots CML1FJPT and CML1FJQR) containing ceftibuten (range tested, 512 to 0.016 μg/mL), ceftibuten-avibactam (range tested, 16/4 to 0.008/4 μg/mL), and ceftazidime-avibactam (range tested, 32/4 to 0.03/4 μg/mL) were prepared by Trek Diagnostic Systems (Thermo Fisher Scientific, Oakwood Village, OH, USA) following CLSI M07 (15) and M100 (16) criteria and distributed to each of the participating laboratories. The 5 quality control (QC) organisms included Escherichia coli ATCC 25922, E. coli NCTC 13353, Klebsiella pneumoniae ATCC 700603, K. pneumoniae ATCC BAA-1705, and K. pneumoniae ATCC BAA-2814.

**TABLE 1:** Investigators and laboratories participating in the CLSI M23 tier 2 QC study

Investigator	Participating laboratory
M. Castanheira	JMI Laboratories, North Liberty, IA
D. Dressel	IHMA, Schaumburg, IL
C. Knapp	Thermo Fisher Scientific, Oakwood Village, OH
C. Pillar	Microbiologics, Kalamazoo, MI
P. Simner	Johns Hopkins, Baltimore, MD
D. Diekema	University of Iowa Hospitals and Clinics, Iowa City, IA
S. Riedel	Beth Israel-Deaconess Medical Center, Boston, MA
D. Snydman	Tufts University Medical Center, Boston, MA

Broth microdilution susceptibility testing was performed according to CLSI M07 (15) and M23 (13) tier 2 criteria. Each laboratory performed susceptibility testing over a minimum of 3 days with no more than 4 replicates tested per day. Each MIC replicate utilized an individually prepared inoculum suspension. Bacterial colony counts (expressed as CFU per milliliter) were performed daily to verify starting inoculum concentrations. A minimum of 5 inoculum verifications per organism per participating laboratory were performed. The average starting inoculum concentrations were 2.3 × 10^5^ CFU/mL for E. coli ATCC 25922, 2.5 × 10^5^ CFU/mL for E. coli NCTC 13353, 2.2 × 10^5^ CFU/mL for K. pneumoniae ATCC 700603, 2.2 × 10^5^ CFU/mL for K. pneumoniae ATCC BAA-1705, and 1.8 × 10^5^ CFU/mL for K. pneumoniae ATCC BAA-2814.

### Data analysis.

In this study, CLSI M23 (13) tier 2 criteria and the RangeFinder statistical program (17) were used to calculate the proposed broth microdilution QC ranges. A 3-dilution QC range was proposed if at least 95.0% of all MIC values (minimum of 210 MIC values from ≥7 participating laboratories) fell within a 3-dilution MIC range. A 4-dilution MIC QC range containing at least 95.0% of all MIC values was proposed (minimum of 210 MIC values from ≥7 participating laboratories) if a bimodal distribution of MIC values was observed or if the height of the second MIC peak was ≥60.0% of the height of the primary MIC peak.

RangeFinder is an Excel-based spreadsheet developed by Turnidge and Bordash (17) containing embedded macros that perform statistical analysis calculations on the broth microdilution MIC data submitted by the laboratories participating in CLSI M23 (13) tier 2 studies. The statistical calculations included mean, median, and modal MIC values as well as the geometric mean. For MIC data from a participating laboratory to be excluded from the overall data analysis for a particular organism/drug combination, at least 2 or more of the calculated statistical values (mean, median, and/or modal MIC) needed to be designated by the RangeFinder program as statistical outliers. In this study, no statistical outliers were identified, and no laboratories were excluded from the data analysis.

### Data availability.

Data will be made available upon reasonable request.

## RESULTS

**FIG 1:**
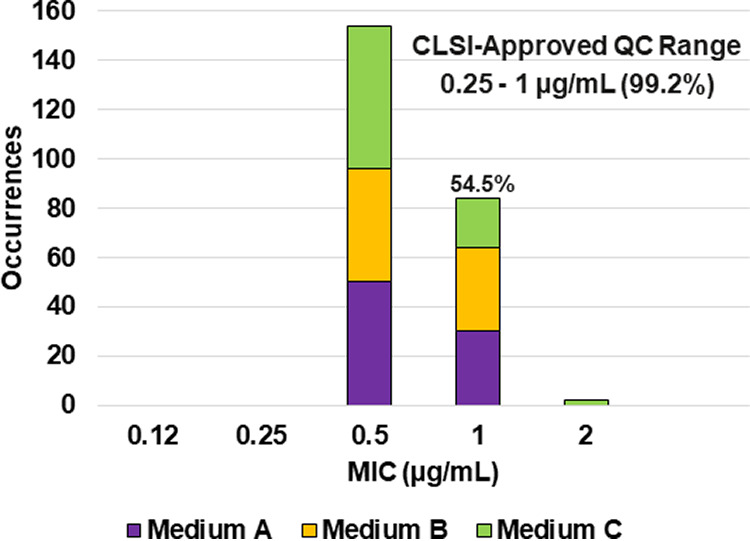
Ceftibuten MIC distributions by medium lot for K. pneumoniae ATCC 700603. Medium A, Difco, lot 9156821; medium B, BBL (BD), lot 0252344; medium C, Oxoid, lot 3163254. A 54.5% ceftibuten MIC shoulder was observed at 1 μg/mL.

**FIG 2:**
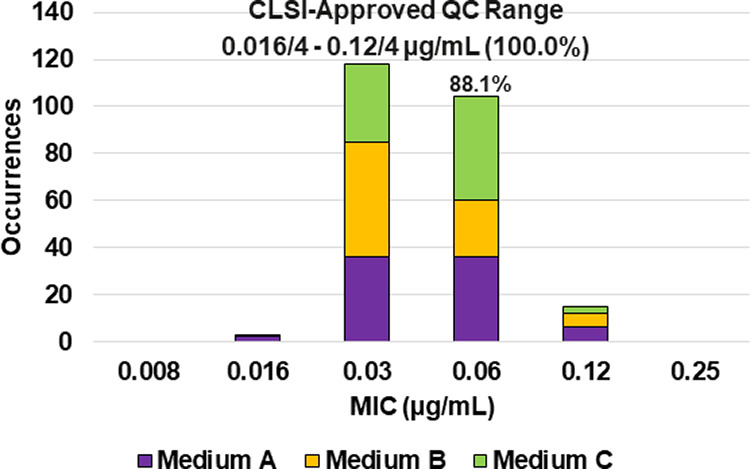
Ceftibuten-avibactam MIC distributions by medium lot for Escherichia coli ATCC 25922. Medium A, Difco, lot 9156821; medium B, BBL (BD), lot 0252344; medium C, Oxoid, lot 3163254. An 88.1% ceftibuten-avibactam MIC shoulder was observed at 0.06/4 μg/mL.

**FIG 3:**
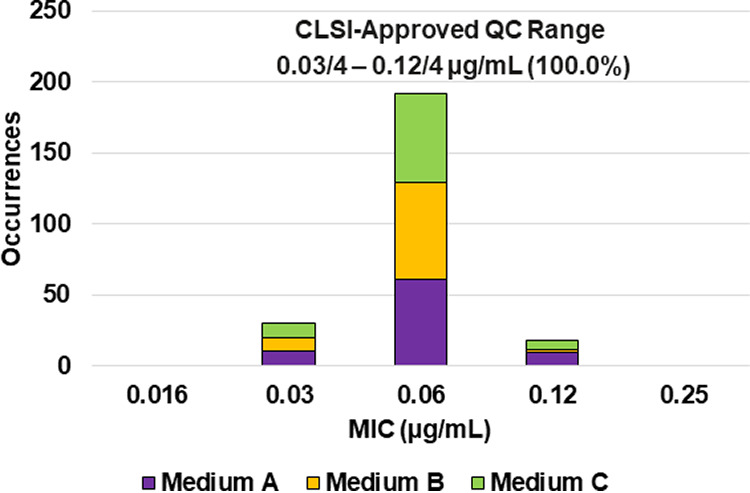
Ceftibuten-avibactam MIC distributions by medium lot for Escherichia coli NCTC 13353. Medium A, Difco, lot 9156821; medium B, BBL (BD), lot 0252344; medium C, Oxoid, lot 3163254.

**FIG 4:**
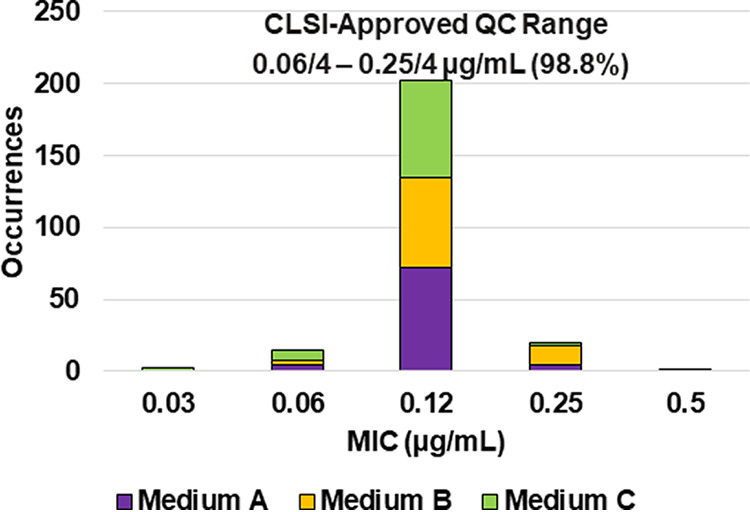
Ceftibuten-avibactam MIC distributions by medium lot for Klebsiella pneumoniae ATCC 700603. Medium A, Difco, lot 9156821; medium B, BBL (BD), lot 0252344; medium C, Oxoid, lot 3163254.

**FIG 5:**
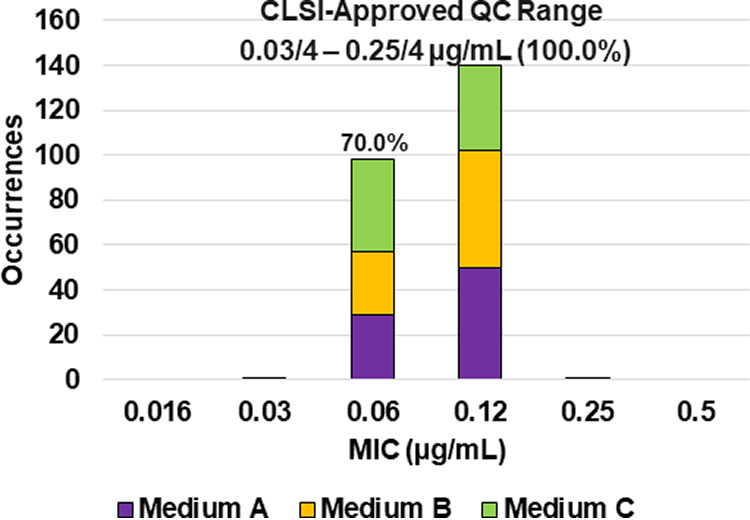
Ceftibuten-avibactam MIC distributions by medium lot for Klebsiella pneumoniae ATCC BAA-1705. Medium A, Difco, lot 9156821; medium B, BBL (BD), lot 0252344; medium C, Oxoid, lot 3163254. A 70.0% ceftibuten-avibactam MIC shoulder was observed at 0.06/4 μg/mL.

**FIG 6:**
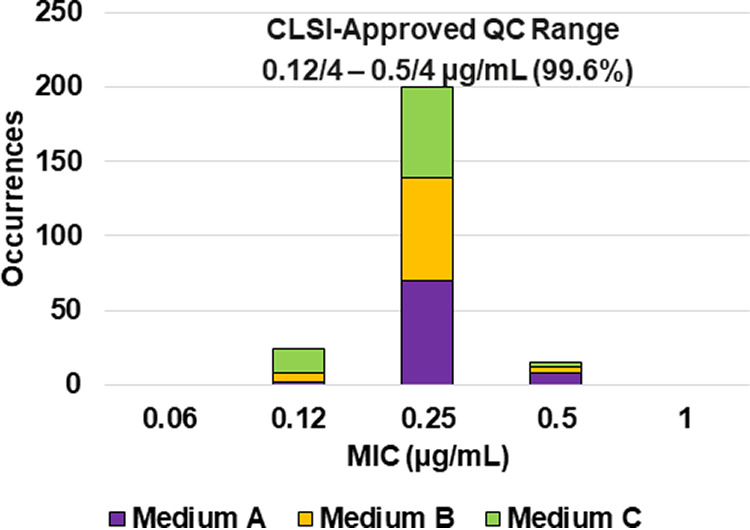
Ceftibuten-avibactam MIC distributions by medium lot for Klebsiella pneumoniae ATCC BAA-2814. Medium A, Difco, lot 9156821; medium B, BBL (BD), lot 0252344; medium C, Oxoid, lot 3163254.

**FIG 7:**
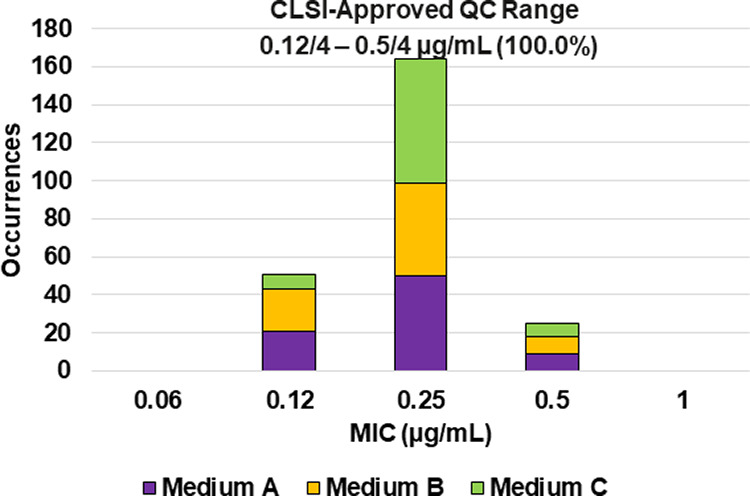
Ceftazidime-avibactam MIC distributions by medium lot for Escherichia coli NCTC 13353. Medium A, Difco, lot 9156821; medium B, BBL (BD), lot 0252344; medium C, Oxoid, lot 3163254.

**FIG 8:**
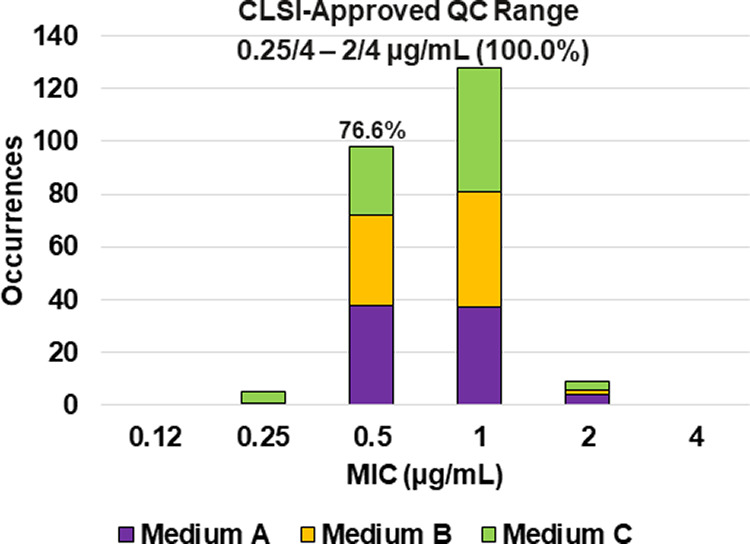
Ceftazidime-avibactam MIC distributions by medium lot for Klebsiella pneumoniae ATCC BAA-1705. Medium A, Difco, lot 9156821; medium B, BBL (BD), lot 0252344; medium C, Oxoid, lot 3163254. A 76.6% ceftazidime-avibactam MIC shoulder was observed at 0.5/4 μg/mL.

**FIG 9:**
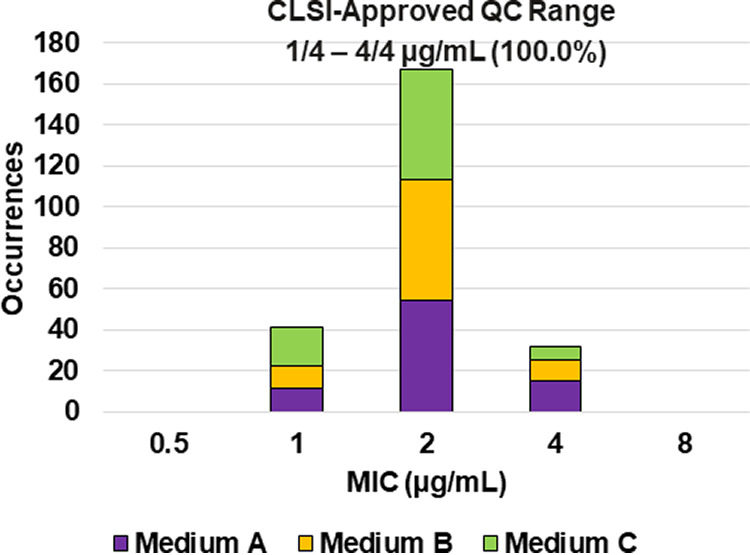
Ceftazidime-avibactam MIC distributions by medium lot for Klebsiella pneumoniae ATCC BAA-2814. Medium A, Difco, lot 9156821; medium B, BBL (BD), lot 0252344; medium C, Oxoid, lot 3163254.

The CLSI M23 (13) tier 2 QC study design provides the opportunity to establish reproducible QC ranges that encompass the variability inherent in antibacterial susceptibility testing while also accounting for differences in microbiology media. The results of this study supported the recent establishment of the ceftibuten, ceftibuten-avibactam, and ceftazidime-avibactam QC ranges as shown in Table 2 and Fig. 1 to 9.

**TABLE 2:** Recently approved CLSI broth microdilution quality control MIC ranges for ceftibuten, ceftibuten-avibactam, and ceftazidime-avibactam

QC strain tested	Recently approved CLSI broth microdilution QC range (μg/mL) (% in range; no. of dilutions)		
	Ceftibuten	Ceftibuten-avibactam (fixed 4 μg/mL)	Ceftazidime-avibactam (fixed 4 μg/mL)
E. coli ATCC 25922		0.016/4–0.12/4 (100.0%; 4)	
E. coli NCTC 13353		0.03/4–0.12/4 (100.0%; 3)	0.12/4–0.5/4 (100.0%; 3)
K. pneumoniae ATCC 700603	0.25–1 (99.2%; 3)	0.06/4–0.25/4 (98.8%; 3)	
K. pneumoniae ATCC BAA-1705		0.03/4–0.25/4 (100.0%; 4)	0.25/4–2/4 (100.0%; 4)
K. pneumoniae ATCC BAA-2814		0.12/4–0.5/4 (99.6%; 3)	1/4–4/4 (100.0%; 3)

### Reference strains with approved QC ranges.

Among the reference bacterial strains tested with approved QC ranges, 100.0% (240/240) of the ceftibuten MIC values against E. coli ATCC 25922 (0.12 to 0.5 μg/mL), E. coli NCTC 13353 (16 to 64 μg/mL), K. pneumoniae ATCC BAA-1705 (4 to 32 μg/mL), and K. pneumoniae ATCC BAA-2814 (8 to 32 μg/mL) were within the QC ranges published in the CLSI M100 (16) document. For ceftazidime-avibactam, 100.0% of the MIC values against E. coli ATCC 25922 (0.06/4 to 0.5/4 μg/mL) and K. pneumoniae ATCC 700603 (0.25/4 to 2/4 μg/mL) were within CLSI-approved QC ranges contained in the M100 (16) document. The ceftibuten and ceftazidime-avibactam MIC QC results provide validated internal controls on each day of susceptibility testing.

### Ceftibuten.

Applying CLSI M23 (13) criteria and the RangeFinder statistical program to establish QC ranges, 99.2% (238/240) of the ceftibuten MIC results against K. pneumoniae ATCC 700603 were within the approved 3-dilution QC range of 0.25 to 1 μg/mL (Table 2 and Fig. 1). A 54.5% ceftibuten MIC shoulder was observed at 1 μg/mL. This MIC shoulder is below the 60.0% threshold required to expand the dilution range to 4 dilutions.

### Ceftibuten-avibactam (fixed 4 μg/mL).

Applying CLSI M23 (13) criteria and the RangeFinder statistical program to establish ceftibuten-avibactam MIC QC ranges, 3-dilution QC ranges were approved for E. coli NCTC 13353 (0.03/4 to 0.12/4 μg/mL), K. pneumoniae ATCC 700603 (0.06/4 to 0.25/4 μg/mL), and K. pneumoniae ATCC BAA-2814 (0.12/4 to 0.5/4 μg/mL) containing 100.0%, 98.8%, and 99.6%, respectively, of all ceftibuten-avibactam MIC values (Table 2 and Fig. 3, 4, and 6). A 4-dilution ceftibuten-avibactam QC range was approved by the CLSI QC working group for E. coli ATCC 25922 (0.016/4 to 0.12/4 μg/mL) and K. pneumoniae ATCC BAA-1705 (0.03/4 to 0.25/4 μg/mL) containing 100.0% of all MIC values (Table 2 and Fig. 2 and 5). The 88.1% and 70.0% ceftibuten-avibactam MIC shoulder values for E. coli ATCC 25922 and K. pneumoniae ATCC BAA-1705 at 0.06/4 μg/mL indicate the need for the 4-dilution QC ranges (Fig. 2 and 5).

### Ceftazidime-avibactam (fixed 4 μg/mL).

Applying CLSI M23 (13) criteria and the RangeFinder statistical program to establish ceftazidime-avibactam broth microdilution QC ranges, 3-dilution QC ranges were approved for E. coli NCTC 13353 (0.12/4 to 0.5/4 μg/mL) and K. pneumoniae ATCC BAA-2814 (1/4 to 4/4 μg/mL) containing 100.0% of all ceftazidime-avibactam MIC values (Table 2 and Fig. 7 and 9). A 4-dilution ceftazidime-avibactam QC range was approved for K. pneumoniae ATCC BAA-1705 (0.25/4 to 2/4 μg/mL) containing 100.0% of all ceftazidime-avibactam MIC values (Table 2 and Fig. 8). The 76.6% ceftazidime-avibactam MIC shoulder at 0.5/4 μg/mL for K. pneumoniae ATCC BAA-1705 indicated the need for the fourth dilution (Table 2 and Fig. 8).

## DISCUSSION

Increasing Gram-negative bacterial resistance to conventional oral antibacterials, including ciprofloxacin, nitrofurantoin, and trimethoprim-sulfamethoxazole, in patients with urinary tract infections has created a medical need for new treatment options (5). Several antibacterial agents and agent combinations with an oral treatment option are currently in clinical development for uncomplicated and/or complicated urinary tract infections including ceftibuten/ARX-1796 (avibactam prodrug), cefpodoxime-ETX0282, ceftibuten/ledaborbactam, ceftibuten-xeruborbactam, sulopenem, and tebipenem. Each of these agents and agent combinations has demonstrated potent *in vitro* antibacterial activity against *Enterobacterales* isolates from patients with urinary tract infections, including extended-spectrum β-lactamase (ESBL)-producing and carbapenem-resistant strains (18–22).

Ceftibuten is an orally active third-generation cephalosporin originally approved by the U.S. FDA in December 2015 for treatment of acute exacerbations of chronic bronchitis, otitis media, pharyngitis, and tonsilitis caused by Haemophilus influenzae, Haemophilus parainfluenzae, Moraxella catarrhalis, and penicillin-susceptible Streptococcus pneumoniae. Tested alone, ceftibuten is active against many Gram-negative pathogens and has demonstrated clinical efficacy in the treatment of uncomplicated and complicated urinary tract infections (23). The addition of the orally active avibactam prodrug (ARX-1796) to ceftibuten expands its Gram-negative antibacterial spectrum to include more recent ESBL (CTX-M)- and carbapenemase (KPC and OXA-48)-producing *Enterobacterales* isolates.

The approved CLSI broth microdilution QC ranges for ceftibuten-avibactam against E. coli ATCC 25922 (0.016/4 to 0.12/4 μg/mL), E. coli NCTC 13353 (0.03/4 to 0.12/4 μg/mL), K. pneumoniae ATCC 700603 (0.06/4 to 0.25/4 μg/mL), K. pneumoniae ATCC BAA-1705 (0.03/4 to 0.25/4 μg/mL), and K. pneumoniae ATCC BAA-2814 (0.12/4 to 0.5/4 μg/mL) will assist both clinical and reference laboratories participating in ceftibuten-avibactam clinical trials and support the regulatory review process for this new antibacterial combination.

In addition, the approved QC ranges for ceftibuten against K. pneumoniae ATCC 700603 (0.25 to 1 μg/mL) and ceftazidime-avibactam against E. coli NCTC 13353 (0.12/4 to 0.5/4 μg/mL), K. pneumoniae ATCC BAA-1705 (0.25/4 to 2/4 μg/mL), and K. pneumoniae ATCC BAA-2814 (1/4 to 4/4 μg/mL) will provide clinical laboratories and device manufacturers with additional options when performing QC testing.
